# Erosive Gastritis and Portal Hypertension

**DOI:** 10.1155/1992/34720

**Published:** 1992

**Authors:** J. G. Geraghty, W. J. Angerson, D. C. Carter

**Affiliations:** University Department of Surgery Royal Infirmary Glasgow United Kingdom

## Abstract

There is conflicting evidence concerning the effects of portal hypertension on the gastric mucosa. This paper summarises the histological and haemodynamic alterations which are present in both human and experimental portal hypertension. Despite the fact that histological studies suggests that the gastric mucosa is an oedematous plethoric structure in portal hypertension, haemodynamic studies show that gastric mucosal blood glow is at least maintained if not increased in portal hypertension. The term “active” rather than “passive” congestion is a more appropriate description of the basic change present in the gastric mucosa in portal hypertension.


*HPB Surgery*, 1992, Vol. 6, pp. 19–22
Reprints available directly from the publisher
Photocopying permitted by license only

© 1992 Harwood Academic Publishers GmbH
Printed in the United Kingdom

### EROSIVE GASTRITIS AND PORTAL HYPERTENSION

J. G. GERAGHTY, W. J. ANGERSON and D. C. CARTER
*University Department of Surgery, Royal Infirmary, Glasgow, United Kingdom*

*(Received 7 January 1992)*

There is conflicting evidence concerning the effects of portal hypertension on the gastric mucosa. This paper summarises the histological and haemodynamic alterations which are present in both human and experimental portal hypertension. Despite the fact that histological studies suggests that the gastric mucosa is an oedematous plethoric structure in portal hypertension, haemodynamic studies show that gastric mucosal blood glow is at least maintained if not increased in portal hypertension. The term "active" rather than "passive" congestion is a more appropriate description of the basic change present in the gastric mucosa in portal hypertension.

KEY WORDS: Gastric, mucosa, portal, hyptertension

Oesophageal varices are the commonest cause of upper gastrointestinal bleeding in patients with hepatic cirrhosis, but there is now an increasing body of evidence suggesting that erosive gastritis may be an important source of bleeding in this patient population[1,2]. It is known that portal hypertension is associated with endoscopically visible abnormalities of the gastric mucosa including a snake skin appearance[3] and cherry red spots[4], and these endoscopic lesions have been proposed as signs diagnostic of portal hypertension. Histological studies of the stomach wall in patients with portal hypertension have shown that mucosal vascular ectasis[5] and submucosal arteriovenous shunts[6] are prominent features and suggest that the microvascular architecture of the stomach is altered in portal hypertension. This concept is strengthened by the finding that animal models of acute pre-hepatic portal hypertension have dilated tortuous submucosal venules associated with marked submucosal oedema[7] and the terms congestive gastropathy and portal hypertensive vasculopathy have been coined to describe this histological change. This body of histological evidence if consistent with the classical concept that raised portal venous pressure is primarily due to the increased resistance to portal venous flow secondary to either hepatic fibrosis or portal vein thrombosis. The proponents of this hypothesis would also argue that portal hypertension is associated with a stagnant gastric microcirculation which renders the gastric mucosa more susceptible to injury and it has been shown in experimental prehepatic portal hypertension that the gastric mucosa is more susceptible to alcohol[7] and bile salt-induced[8] injury.

The concept that the stomach is a passively congested organ in portal hypertension is in conflict, however, with haemodynamic studies which show that both clinical and experimental portal hypertension is associated with a hyperdynamic systemic circulatory state[9–11]. It is also known that this increase in cardiac output is associated with a marked increase in both splanchnic inflow and total gastric blood flow in experimental portal hypertension[12–14]. Given that large increases in gastric mucosal blood flow can reduce mucosal susceptibility to injury[15] these haemodynamic studies suggest that the gastric mucosa in portal hypertension may not be more prone to ulceration. The vast majority of studies examining the effects of portal hypertension on the gastric mucosa have been performed in experimental animal models of prehepatic portal hypertension and the divorce between the results of histological and haemodynamic studies may be linked to the fact that this model is associated with marked temporal changes in splanchnic haemodynamics after portal vein ligation. Sikuler and colleagues have shown that the initial period after portal ligation is associated with a marked reduction in splanchnic inflow but that reversal to a hyperdynamic splanchnic circulatory state is complete seven days after portal vein ligation[16]. It is also known that the level of gastric mucosal perfusion is markedly reduced in acute prehepatic portal hypertension, but that this reduction is not sustained in chronic prehepatic portal hypertension[17] despite the maintenance of an elevated portal pressure in this animal model[18]. Furthermore, experimental studies have shown that the susceptibility to taurocholate-induced injury is not increased in chronic prehepatic portal hypertension[19].

Studies in experimental cirrhosis, which may be more relevant to the clinical situation, are more difficult to perform mainly due to the problems associated with developing animal models of decompensated cirrhosis. The evidence available, however, is unanimous that both total gastric blood flow[12], and gastric mucosal flood flow[20,21] are increased in hepatic cirrhosis. It has also been shown that experimental cirrhosis does not render the gastric mucosa more prone to injury by bile salts[22] -- the bias, if anything, favours a reduced susceptibility to injury in association with documented increases in gastric mucosal blood flow. The main conclusion to be drawn from this body of experimental evidence is that gastric mucosal susceptibility to injury may be more closely linked to the effects of portal hypertension on gastric mucosal blood flow rather than to the presence of raised portal pressure in isolation. These studies also suggest that the term "active" rather than "passive" congestion is a more appropriate description of the basic haemodynamic change affecting the gastric mucosa in portal hypertension. The histological and haemodynamic limbs of the debate are not necessarily in conflict as vascular ectasia, dilated tortuous submucosal arterioles, gastric red spots and submucosal arterio-venous shunts are compatible with the presence of a hyperdynamic circulatory state.

These experimental studies therefore suggest that patients with uncomplicated chronic portal hypertension are not more susceptible to gastric mucosal erosion formation, but that an acute rise in portal venous pressure may increase gastric mucosal susceptibility to injury. Clinical studies fail to give a clear cut answer to this question as the incidence of erosive gastritis as a cause of upper gastrointestinal bleeding in patients with portal hypertension ranges from 10–59% [23,1]. This wide incidence range is not surprising given that complications of hepatic cirrhosis such as jaundice[24], acidosis[25] and hypoxia[26] have a well known association with gastric mucosal erosions.

It is also possible that haemorrhagic shock due to variceal bleeding may cause gastric mucosal stress ulceration or that mucosal ulceration may be a reperfusion injury; these factors may, in part, explain the frequent co-existence of bleeding varices and gastric mucosal erosions[2,27,28]. Such conjecture requires to be tested under experimental conditions. It is clear, however, that raised portal pressure does not necessarily equate with gastric mucosal barrier disruption and the concept that portal hypertension is associated with a stagnant mucosal microcirculation and increased gastric mucosal susceptibility to injury needs revision.

### *References*

1. McCray, R. S., Martin, F., Amir-Ahmadi, H., Sheahan D. and Zamcheck N. (1969) Erroneous diagnosis of haemorrhage from oesophageal varices. *Am. J. Dig. Dis.*, **14**, 755–760
2. Sarfeh, I. J., Tabak, C., Engene, J. and Juler, G. L. (1981) Clinical significance of erosive gastritis in patients with alcoholic liver disease and upper gastrointestinal haemorrhage. *Ann. Surg.*, **194**, 149–151
3. Papazian, A., Braillon, A., Dupas, J. L. *et al.* (1986) Portal hypertensive gastric mucosa: an endoscopic study. *GUT*, **28**, 1199–1203
4. McCormack, T. T., Sims, J., Eyre-Brook, I., *et al.* (1985) Gastric lesions in portal hypertension: gastritis or congestive gastropathy. *GUT*, **26**, 1226–1272
5. Quintero, E., Pique, J. M., Bombi, J. A. *et al.* (1987) Gastric mucosal vascular ectasias causing bleeding in cirrhosis. *Gastroenterology*, **93**, 1054–1061
6. Hashizume, M., Tanaka, K. and Inokuchi, K. (1983) Morphology of gastric microcirculation in cirrhosis. *Hepatology*, **3**, 1008–1012
7. Sarfeh, I. J., Tarnawski, A., Malki, A., Mason, G. R., Mach, T. and Ivey K. J. (1983) Portal hypertension and gastric mucosal injury in rats. *Gastroenterology*, **84**, 987–993
8. Sarfeh, I. J., Tarnawski, A., Maeda, R., Raymont, K., Mason, G. R. and Ivey, K. J. (1984) The gastric mucosa in portal hypertension: Effects of topical bile acid. *Scand. J. Gastroenterol.*, **19**, 189–194
9. Kowalski, H. J. and Abelmann W. H. (1953) The cardiac output at rest in Laennec's cirrhosis. *J. Clin. Invest.*, **32**, 1025–1033
10. Murray, J. F., Dawson, A. M. and Sherlock, S. (1958) Circulatory changes in chronic liver disease. *Am. J. Med.*, **24**, 358–367
11. Vorobioff, J., Bredfeldt, J. E., Groszmann, R. J. (1983) Hyperdynamic circulatory in portal hypertensive rat model: a primary factor for maintenance of chronic portal hypertension. *Am. J. Physiol.*, **244**, 652–657
12. Vorobioff, J., Bredfeldt, J. E. Groszmann, R. J. (1984) Increased blood flow through the portal system in cirrhotic rats. *Gastroenterology*, **87**, 1120–1126
13. Groszmann, R. J., Vorobioff, J. and Riley, E. (1982) Splanchnic haemodynamics in portal hypertensive rats: measurement with gamma-labelled microspheres. *Am. J. Physiol.*, **242**, G156–G160
14. Blanchet, L. and Lebrec, D. (1982) Changes in splanchnic blood flow in portal hypertensive rats. *Eur. J. Clin. Invest.*, **12**, 1002–1007
15. Moody, F. G., McGreevy, J. M., Zalewsky, C., Cheung, L. Y. and Simons, M. (1977) The cytoprotective effect of mucosal blood flow in experimental erosive gastritis. In gastric ion transport proceedings of a symposium on gastric ion transport in Uppsala, Sweden, July 24–28, 1977
16. Sikuler, E., Kravetz, D. and Froszmann, R. J. (1985) Evolution of portal hypertension and mechanisms involved in its maintenance in a rat model. *Am. J. Physiol.*, **248**, G617–G625
17. Geraghty, J. G., Angerson, W. J. and Carter D. C. (1989) An autoradiographic study of the regional distribution of gastric blood flow in portal hypertensive rats. *Gastroenterology*, **97**, 1108–1114
18. Geraghty, J. G. Angerson, W. J. Carter, D. C. (1989) Portal venous pressure and portasystemic shunting in experimental portal hypertension. *Am. J. Physiol.*, **257**, G52–57
19. Angerson, W. J., Geraghty, J. G., Carter, D. C. (1992) Taurocholate-induced gastric mucosal injury in experimental portal hypertension. *GUT* **33**, 170–174
20. Manabe, T., Suzuki, T. and Honjo, I. (1978) Changes of gastric blood flow in experimentally induced cirrhosis of the liver. *Surg. Gynec. Obs.*, **147**, 753–757
21. Geraghty, J. C., Angerson, W. J. and Carter, D. C. A study of regional gastric mucosal blood flow in a rat model of hepatic cirrhosis. *Am. J. Physiol.*, (In press)
22. Geraghty, J. G., Angerson, W. J. and Carter, D. C. (1988) Gastric haemodynamics and susceptibility to injury in portal hypertension. *Gastroenterology*, **94**, A541
23. Waldram, R., Davis, M., Nunnerley, H. and Williams, R. (1974) Emergency endoscopy after gastrointestinal haemorrhage in 50 patients with portal hypertension. *B.M.J..*, **4**, 94–96

24. Mullane, J. F., Ritchie, W. P., Solis, R. T. *et al.* (1972) Experimental biliary obstruction and stress ulcer formation. *J. Surg. Res.*, **12**, 180–184
25. Mullane, J. F., Wilfong, R. G., Phelps, T. O. and Fischer, R. P. (1973) Metabolic acidosis, stress, and gastric lesions in the rat. *Arch Surg.*, **107**, 456–459
26. Skillman, J. J., Bushnell, L. S., Goldman, H. and Silen, W. (1969) Respiratory failure, hypotension, sepsis and jaundice: a clinical syndrome associated with lethal haemorrhage from acute stress ulceration of the stomach. *Am. J. Surg.*, **117**, 523–530
27. Franco, D., Deporte, A., Durandy, Y. and Bismuth, H. (1977) Upper gastrointestinal haemorrhage in hepatic cirrhosis: causes and relation to hepatic failure and stress. *Lancet,* **1**, 218–220
28. Thomas, E., Rosenthal, W. S., Rymer, W. and Katz, D. (1979) Upper gastrointestinal haemorrhage in patients with alcoholic liver disease and oesophageal varices. *Am. J. Gastroenterol.,* **72**, 623–629

*<Accepted by S. Bengmark, 13 January 1992)*

